# Adsorption and movement of water by skin of the Australian thorny devil (Agamidae: *Moloch horridus*)

**DOI:** 10.1098/rsos.170591

**Published:** 2017-09-13

**Authors:** Philipp Comanns, Falk J. Esser, Peter H. Kappel, Werner Baumgartner, Jeremy Shaw, Philip C. Withers

**Affiliations:** 1 Institute of Biology II, RWTH Aachen University, Worringerweg 3, 52074 Aachen, Germany; 2 Botanischer Garten, Plant Biomechanics Group Freiburg, University of Freiburg, Schänzlestraße 1, 79104 Freiburg, Germany; 3 Institute of Biomedical Mechatronics, Johannes Kepler University Linz, Altenberger Strasse 69, 4040 Linz, Austria; 4 Centre for Microscopy, Characterisation and Analysis (CMCA), University of Western Australia, 35 Stirling Highway, Crawley, Western Australia 6009, Australia; 5 School of Biological Sciences, University of Western Australia, 35 Stirling Highway, Crawley, Western Australia 6009, Australia

**Keywords:** thorny devil, water transport, skin, capillary, distance extension, biomimetic

## Abstract

Moisture-harvesting lizards, such as the Australian thorny devil *Moloch horridus*, have remarkable adaptations for inhabiting arid regions. Their microstructured skin surface, with channels in between overlapping scales, enables them to collect water by capillarity and passively transport it to the mouth for ingestion. We characterized this capillary water transport for live thorny devils using high-speed video analyses. Comparison with preserved specimens showed that live lizards are required for detailed studies of skin water transport. For thorny devils, there was no directionality in cutaneous water transport (unlike *Phrynosoma*) as 7 µl water droplets applied to the skin were transported radially over more than 9.2 mm. We calculated the total capillary volume as 5.76 µl cm^−2^ (dorsal) and 4.45 µl cm^−2^ (ventral), which is reduced to 50% filling by the time transportation ceases. Using micro-computed tomography and scanning electron microscopy of shed skin to investigate capillary morphology, we found that the channels are hierarchically structured as a large channel between the scales that is sub-divided by protrusions into smaller sub-capillaries. The large channel quickly absorbs water whereas the sub-capillary structure extends the transport distance by about 39% and potentially reduces the water volume required for drinking. An adapted dynamics function, which closely reflects the channel morphology, includes that ecological role.

## 1. Introduction

Adaptations in nature to limited resources such as water scarcity are well studied. Some snakes, toads, arthropods and even mammals have been found to survive water scarcity by using their body surface to collect water from various sources [1–7]. Several plants also show water collection mechanisms [8–10]. Collection of water and/or water handling has been of particular interest regarding the potential transfer of the underlying water collection mechanisms to technical applications, e.g. for fog collection [11–14], condensation [12,15] or water transportation [7,16]. Some specialized lizards, like the Australian thorny devil (Agamidae: *Moloch horridus*) and the Texas horned lizard (Iguanidae: *Phrynosoma cornutum*), have a spectacular ability to harvest environmental moisture using their skin [17,18]. These lizards can inhabit arid environments presumably in part because of their ability to collect water on their skin when drinking water is inaccessible [19–25]. The phenomenon of collecting environmental water after rain fall using the skin has previously been termed ‘water absorption’ [26,27], ‘blotting paper effect’ [28] and ‘rain-harvesting’ [21,22,29]. The recently proposed term ‘moisture harvesting’ is perhaps the most appropriate term, as it describes the different kinds of water acquisition more comprehensively [19]. Such moisture-harvesting systems have interesting biomimetic implications. Accordingly, biomimetic studies of these lizards are invaluable, with respect to adapting biological mechanisms for anthropological roles of water acquisition e.g. for surface wettability and directional liquid transport [19,30], sampling/collection of target material for detection systems [31], or lubrication [32].

The physical ability of thorny devils and horned lizards to harvest moisture is apparently required by their inability to drink directly, reflecting their oral and lingual adaptations for eating small ants [18,33,34]. Water collection is enabled by the role of small skin channels between the scales that accumulate water, which then is transported to the mouth for drinking [19,20,23,35]. In particular, the channels enable a passive transport of collected water to the mouth by capillary action [19,23,25], where (active) ingestion occurs by jaw movements [22,25,35]. As the integument of desert lizards is substantially waterproof to minimize water loss by evaporation [25,28,36], adsorption of collected water across the skin is precluded. Thus transportation of water to the mouth is a physiologically essential requirement.

The skin channels that facilitate water transport are formed by partially overlapping (imbricate) scales with a narrow opening on their superficial side by which a semi-tubular system of channels is formed that extends over the lizard's entire body surface [23]. In morphological studies the channels were found to have a width of 100–250 µm at their basal part, but narrower openings at the surface, i.e. 100–150 µm [23,25]. Besides these morphological investigations, an experimental approach has predicted an average channel width of 224 µm, from equilibrium of the capillary force and gravitational force at 9.9 cm vertical transport distance [25]. The channels have globular protrusions that form narrow sub-channels of up to 50 µm depth and their surface is covered with an Oberhäutchen surface structure, i.e. fine-scale dimpling [23,35].

Previous skin morphology studies have focused on single channels or described their general role in water transport. Consequently, we characterize here the channel structures and how they transport water over the skin in more detail, regarding water velocity, transport distance and directionality of applied droplets for live thorny devils. We also compare water transport functionality with morphological aspects of the capillary network, with a focus on determining the potential role of the sub-channels in the skin for water transport. Water transport by skin capillaries is also analysed for preserved thorny devils (museum specimens) to determine whether they can substitute for experiments with live lizards, and be used to screen museum collections for further species with the potential ability of skin water transport. Our findings are also applied to a model of skin water transport to illustrate the functional principles of the skin micro-structural adaptation of *M. horridus* to its ecological niche.

## 2. Material and methods

*Moloch horridus* (snout–vent length: 94.5 ± s.e.m. 4.7 mm, body weight: 39.86 ± 6.39 g; *n* = 6) were collected at Mt. Gibson Iron Ltd. mine site (Western Australia) and transported to the laboratory at the University of Western Australia. They were maintained in terraria with sand substrate and vegetation for environmental enrichment, and fed locally-collected ants every 1–2 days; they were returned to their capture site after 18 days. Each lizard was used in experiments less often than every second day, and only one experiment was conducted per lizard per day to minimize any handling stress. Thorny devils were weighed to ±0.0001 g using an A&D electronic balance (ER-182A).

### 2.1. Cutaneous water transport

We applied 7 µl droplets of coloured water (1% blue food dye, Queen Fine Foods, Alderley, Australia) onto the skin of live thorny devils, and video recorded the adsorption and spread of the water droplet while lizards were gently held in position by hand for up to one minute. The dyed water was washed off the skin immediately after video-recording stopped.

Skin capillary water transport was also analysed for preserved specimens (Western Australian Museum, *Moloch horridus* 124889, 164322, 166290, 166291). The preserved lizards (stored in 70% ethanol) were dried in air for one hour before coloured water droplets were applied and video recorded. All measurements were repeated on a second day.

Videos of the transported coloured water droplets provided an optical method for velocity measurement. Video analysis was conducted using digital high-speed microscopy (125 fps; Keyence VW-9000, Keyence Deutschland GmbH, Neu-Isenburg, Germany) with accompanying analysis software. For scale, the video image was calibrated for each recording using a ruler placed on the lizard skin before applying the water droplet (time *t*_0_). The number of channels and spreading area when the water droplet was no longer apparent above the surface (time *t*_1_) and when water transport ceased (time *t*_2_) were determined from droplets applied to each thorny devil's dorsal and ventral body surface (i.e. one droplet per lizard per surface). As the channels are open toward the skin surface, coloured water remains visible in the channels throughout the experiment. We then measured the spreading area for the droplets and calculated the number of channels for *t*_1_ and *t*_2_ according to the previously determined channel density.

### 2.2. Morphological scanning electron microscopy analyses

Moulting skin samples were also used for scanning electron microscope (SEM) imaging. The shed skin was sputter-coated (without further treatment) with gold and examined using a SEM (525 M; Philips AG, Amsterdam, Netherlands). The length of skin channels was determined as the mean distance between the intersection points with two other channels. The precise location of an intersection between two channels was defined as the geometric centre of a circle that was accurately placed in the intersection area.

### 2.3. Morphological light microscopy analyses

Moulting skin samples were gently removed from dorsal and ventral body sides of a specimen preserved when about to shed (Zoological Research Museum Koenig, Bonn, Germany, *Moloch horridus* 21313) using polyvinysiloxane (President light body casting material, Coltene, Switzerland). As topological negatives, they enabled visualization of the channel network. Light microscopic analysis of the skin morphology was conducted using a binocular microscope (SMZ-168, Motic Ltd., Xiamen, China) with a digital camera (Moticam 3.0, Motic Ltd., Xiamen, China).

### 2.4. Morphological micro-computed tomography analyses

For analysis of the skin channels by micro-computed tomography (µCT), skin samples (approx. 1 × 3 mm) were taken from different body regions of an alcohol-fixed specimen (Western Australian Museum, Perth, Australia, *Moloch horridus* 124889). These samples were fixed in position in a polyethylene tube. A negative stain containing 350 mg organically-bound iodine per ml (Optiray® 350, Tyco Healthcare, Cork, Ireland) was used to provide contrast with the fluid surrounding the scales. The polyethylene tube was then sealed with dental wax and scanned by µCT at 40 keV and 75 µA (Versa 520, Zeiss) with 4× optical magnification. A scan resolution of 1.5 µm pixel size was achieved with a scan volume of 1.5 mm^3^. A total of 1601 projection images were acquired through 360° rotation, with an exposure time of 6 s each. A camera binning of 2 was used to reduce background noise. The raw data were reconstructed (XMReconstructor v8, Zeiss) using a centre shift of −2 and standard beam hardening correction with a beam hardening constant of 0.5 and a reconstruction filter set to a kernel size of 0.7. Reconstructed axial sections were saved as 16 bit TXM files and visualized using a 3D software package (Avizo for FEI Systems – Materials Science v. 8.1.0, FEI).

### 2.5. Statistical analyses

All data are presented as mean ± s.e.m. (with sample size *n*) unless indicated otherwise. Data were analysed using a Student's *t*-test or analysis of variance (ANOVA) followed by a least significant difference (LSD) test for multiple comparisons between pairs of means. Non-parametric tests such as Wilcoxon Signed Rank test or Kruskal–Wallis test were used to avoid assumptions of underlying distributions of the data. All tests were performed using SPSS v. 21.0 (IBM); *p* < 0.05 was regarded as significant.

## 3. Results

### 3.1. Capillary water transport for live thorny devils

**Figure 1.**
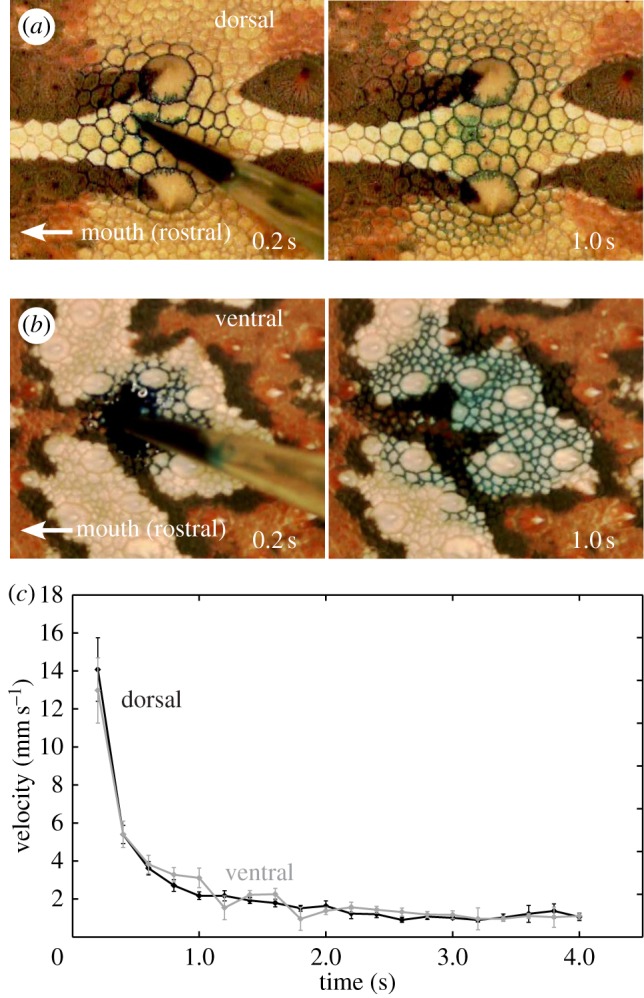
Capillary water transport of 7 µl droplets on the skin of *Moloch horridus*. (*a*) Dorsal surface. (*b*) Ventral surface. (*c*) Velocity on different body sides. Error bars indicate s.e.m.; *n* = 22 (dorsal), *n* = 19 (ventral).

Coloured water droplets applied to the skin of live thorny devils rapidly entered the skin channels and spread over the skin surface in all directions from the point of application (figure 1*a*,*b*). The transport velocity of the coloured water droplet decreased rapidly over time (figure 1*c*; electronic supplementary material, videos S1 and S2). Both dorsal and ventral skin had similar velocities of spreading, independent of rostral, caudal and lateral directions (electronic supplementary material, figure S3). When comparing the mean velocities in all directions for dorsal and ventral skin, the initial velocities appear to be slightly faster dorsally (14.08 ± 1.76 mm s^−1^) than on the ventral body side (11.53 ± 1.65 mm s^−1^).

Water droplets applied to the lizards' skin at time *t*_0_ were adsorbed completely (i.e. at *t*_1_) into the channels between the scales after 1.17 ± 0.35 s (dorsal) and 2.08 ± 0.38 s (ventral). Transportation then continued significantly (repeated measures ANOVA: *F*_1,36_ = 93.2, *p* < 0.001) until *t*_2_, the time by which velocities became un-measurable and the water front appeared to stop at maximum duration. The transport duration time *t*_2_ for live thorny devils was 9.79 ± 0.91 s (dorsal) and 8.56 ± 0.98 s (ventral).There were no significant difference between body sides (repeated measures ANOVA: *F*_1,36_ = 0.03, *p* = 0.856).

A radial transportation was observed for water droplets (7 µl) applied to live thorny devils' skin, i.e. the same distance in every direction (ANOVA: *F*_2,249_ = 0.39, *p* = 0.681), but most of the scale surface did not become wetted (figure 1*a*,*b*). We therefore determined the average transport distance as mean radius of rostral, caudal and lateral directions, which at time *t*_1_ were 6.36 ± 0.16 mm for dorsal and 6.97 ± 0.27 mm for ventral skin, respectively. These radii are significantly smaller than those measured at time *t*_2_ for dorsal (9.20 ± 0.22 mm) and for ventral (9.25 ± 0.25 mm) skin (repeated measures ANOVA: *F*_1,124_ = 6.55, *p* = 0.012). No significant differences were obtained between body sides (repeated measures ANOVA: *F*_1,124_ = 1.14, *p* = 0.288).

The skin area covered by 7 µl droplets was measured as 121.5 ± 6.6 mm^2^ (dorsal) and 157.4 ± 17.7 mm^2^ (ventral) at *t*_1_, and as 268.5 ± 15.2 mm^2^ (dorsal) and 289.5 ± 27.6 mm^2^ (ventral) at *t*_2_. There was a significant difference between *t*_1_ and *t*_2_ (repeated measures ANOVA: *F*_1,39_ = 196.2, *p* < 0.001), but not between dorsal and ventral sides (repeated measures ANOVA: *F*_1,39_ = 1.65, *p* = 0.207). To determine the channel volume per area, we first determined the total number of included skin channels at *t*_2_ for dorsal and ventral body side with one droplet per thorny devil, i.e. six in total. The resulting channel density was 5.95 ± 1.02 mm^−2^ (dorsal) and 5.44 ± 0.80 mm^−2^ (ventral), which did not differ significantly (*t*-test: *p* = 0.679). Based on these reference values we calculated the number of included channels from measured transportation areas as 722.5 ± 39.2 (dorsal) and 856.4 ± 96.4 (ventral) at *t*_1_, and as 1596.5 ± 90.4 (dorsal) and 1575.0 ± 150.1 (ventral) at *t*_2_. Again, *t*_1_ and *t*_2_ differed significantly (repeated measures ANOVA: *F*_1,39_ = 201.0, *p* < 0.001), but no significant difference was obtained between body sides (repeated measures ANOVA: *F*_1,39_ = 0.21, *p* = 0.651). The channel volume was then calculated from both the number of channels at the time *t*_1_ and the transportation area as 5.76 µl cm^−2^ (dorsal) and 4.45 µl cm^−2^ (ventral).

### 3.2. Capillary water transport for preserved thorny devils

For thorny devils preserved as museum specimens, water transport was less pronounced than for live specimens, indicated by a delay of up to several seconds after application of the droplet to the start of transport, and longer transport durations up to 105 s (table 1). However, the delay was not significantly different from zero (one-sample Wilcoxon Signed Rank test: *p*_(dorsal)_ = 0.068, *p*_(ventral)_ = 0.317, *p*_(head)_ = 0.109). The subsequent transport duration was shortest for ventral skin and longest for dorsal skin, whereas duration for lateral head skin was intermediate (table 1).

**Table 1.** Capillary transport of coloured water droplets by the skin of preserved specimens of *Moloch horridus*. Values are given as mean ± s.e.m.; number of analysed videos is given in brackets. A Kruskal–Wallis test was used to test for potential differences of the medians.

	dorsal (6)	ventral (3)	lateral head (6)	significance
duration (s)	29.5 ± 15.3	11.2 ± 5.4	13.1 ± 3.5	$\chi_{\left(2\right)}^{2}=0.692$; *p* = 0.708
delay (ms)	780.0 ± 475.9	34.7 ± 34.7	42.7 ± 20.1	$\chi_{\left(2\right)}^{2}=3.074$; *p* = 0.215

### 3.3. Morphology of skin channels

**Figure 2.**
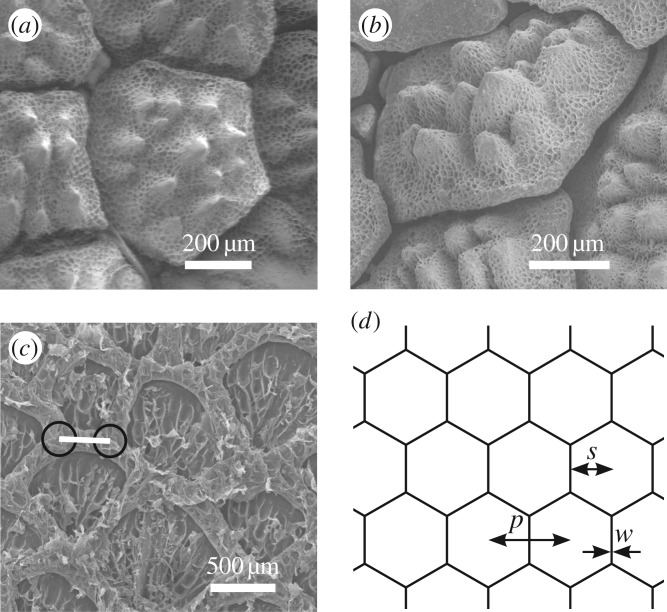
Skin morphology of *Moloch horridus* with capillary channels in between the scales. (*a*) Dorsal scale topography by SEM imaging. (*b*) Overlapping of dorsal scales. (*c*) Inner side of ventral exuviae. The length of capillaries (white bar) was determined as the mean distance between the intersection points with two other capillaries (geometric centre of a circle (here: black circle) placed in the intersection area). (*d*) Scheme of hexagonal capillary network structure. The values for modelling are indicated; pitch *p*, scale radius *s*, width of channel *w*.

Single water droplets were distributed over the lizards' skin immediately after application. Water adsorption and transport occurred mainly within the channels between the overlapping scales (figure 1*a*,*b*) and the scale surface remained un-wetted. The channel cavity is open towards the skin surface (figure 2*a*,*b*), and transported water is visible when the channels are filled or adjacent parts of the scales are wetted. We used shed skin layers to measure morphological parameters such as network structure, channel length and width, using their topological negatives. The channels encircle irregularly hexagonal scales with large spines, so the broad channel network has an almost hexagonal structure (figure 2*c*,*d*). In this hexagonal arrangement the longitudinal oriented channels had a length of 366.9 ± 15.5 µm (dorsal) and 485.8 ± 20.3 µm (ventral), and were significantly longer than laterally oriented channels (dorsal: 308.9 ± 15.2 µm, ventral: 284.4 ± 18.9 µm; *t*-tests: *p* < 0.017) (table 2). A significant difference between dorsal and ventral sides was only found for longitudinal channels, i.e. in length (*t*-tests: *p*_(long)_ < 0.001; *p*_(lateral)_ = 0.315), whereas the width was significantly larger for the dorsal side (186.0 ± 6.6 µm) than ventral side (158.3 ± 5.3 µm; *t*-tests: *p*_(long)_ = 0.003; *p*_(lateral)_ = 0.562) (table 2).

**Table 2.** Length and width of capillary channels. Values are given as mean ± s.e.m. (*n*).

	longitudinal	lateral	*t*-test
	*length of capillaries* (µm)		
dorsal	366.9 ± 15.5 (51)	308.9 ± 15.2 (27)	*p* = 0.017
ventral	485.8 ± 20.3 (34)	284.4 ± 18.9 (19)	*p* < 0.001
*t*-test	*p* < 0.001	*p* = 0.315	
	*width of capillaries* (µm)		
dorsal	186.0 ± 6.6 (51)	189.4 ± 8.8 (27)	*p* = 0.761
ventral	158.3 ± 5.3 (34)	197.2 ± 9.9 (19)	*p* < 0.001
*t*-test	*p* = 0.003	*p* = 0.562	

**Figure 3.**
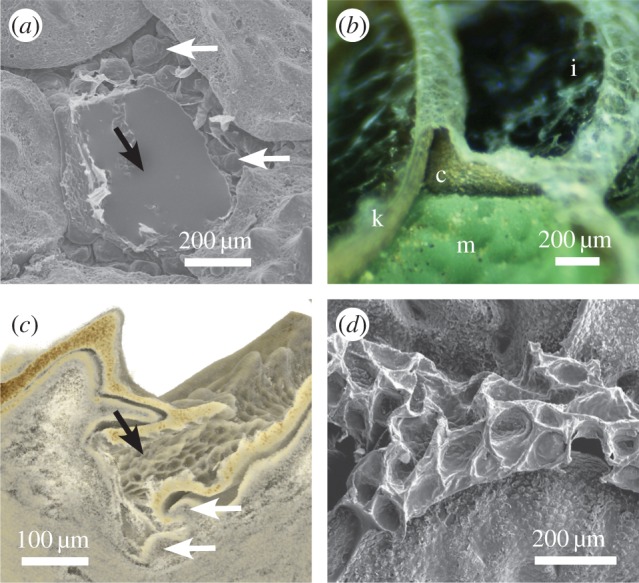
Protrusions in capillary channels of *Moloch horridus*. (*a*) Top view on channels surrounding removed scale (black arrow). Protrusions (white arrows) reach into the channel cavity. (*b*) Light microscopy of shed skin on casting material (m). The channel cavity (c) formed by a thin keratin layer (k) of the exuviae is well preserved. Orientation of exuviae is indicated by inner side (i). (*c*) Cross section through skin using µCT. The microstructure extends to the channel surface (black arrow) and protrusions (white arrows) reach into the channel cavity. (*d*) SEM imaging of skin cast from ventral side, i.e. negative form of skin channel topology.

Removing single scales from skin samples allowed the bottom of the channels to be visible. Here, numerous protrusions of varying size and shape are found (figure 3*a*). However, such extensions into the channel cavity were only found at their bottom (figure 3*b*,*c*). Micro-computer tomographic measurements revealed an irregular shape of the channels with their surface covered by the same micro-ornamentation (Oberhäutchen) as the scales (figure 3*c*). Skin casts of the channels give the negative skin topography and the protrusions appear as numerous elliptical or round shaped inversions (figure 3*d*). This was used to measure size parameters of the protrusions; their diameter varied between 30 µm and 150 µm. The distance between two protrusions, i.e. the ‘partial wall’ formed between two inversions, was considered to be a sub-channel; its size was significantly smaller for dorsal skin (19.1 ± 1.1 µm) than ventral skin (25.1 ± 1.5 µm; *t*-test: *d*_f_ = 78, *p* = 0.002).

### 3.4. Modelling of water transport

To evaluate whether the measured channel parameters are sufficient to describe the observed dynamics of water transport depicted in figure 1, we tested different mathematical models to fit our data for the skin water transport. The measured velocities could not be fitted using a Washburn dynamics function as velocities were generally overestimated. Recently, Chandra & Yang [37] described a dynamic function that contributes more to surface structures, hence friction forces. Besides the measured channel morphometric values, only the contact angle within the channels and the channel depth needed to be modelled. In a first step, the complex skin morphology was abstracted to a structured surface, containing hexagonal pillars (i.e. scales) surrounded by a network of capillary channels. The transport distance of single water droplets becomes dimensionless when divided by the initial droplet radius: *λ* = (*R* – *R*_0_)/*R*_0_. According to this simplification, the measured velocities were modelled with a dynamics function as follows:

3.1 $${\left(1+\lambda \right)}^{2}\text{ln}(1+\lambda )-\frac{\lambda^{2}+2\lambda }{2}=\tau ,$$

with the dimensionless transport distance *λ* and dimensionless time *τ*. Solving equation (3.1) for time *t* yields:

3.2 $$\left({\left(1+\lambda \right)}^{2}\text{ln}(1+\lambda )-\frac{\lambda^{2}+2\lambda }{2}\right)\left(\frac{\kappa \;\eta \;R_{0}^{2}}{2\;h\;\gamma \;(\cos\theta (r-\phi )-1+\phi )}\right)=t,$$

where *h* is the capillary height, *r* the roughness factor (i.e. ratio of actual surface area to its projection), *ϕ* the fraction of the horizontally projected area covered by the scale basis, and *R*_0_ the initial radius of the applied droplet. Some of these geometrical parameters are summarized as *κ* = 1 + *h*^2^/*p*^2^ ln(*p*/*s*), with the average centre-to-centre distance (pitch) *p* and the scale radius *s* (i.e. inner diameter of hexagonal scale basis). The geometrical parameters were determined prior to modelling (table 3, figure 2), and contact angle *θ* and fraction of projected scale area *ϕ* were used as variable fit parameters.

**Table 3.** Parameters determined for modelling the skin water transport using equation (3.2) [37]. Except for *h*, all values were measured from SEM images or light microscopic images (cf. figures 3 and 4).

	dorsal	ventral
scale radius *s* (mm)	0.306	0.325
pitch *p* (mm)	0.798	0.822
initial droplet radius *R*_0_ (mm)	1.0	1.0
roughness factor *r* (−)	1.1660	1.1665
fraction projected scale area *ϕ* (−)	0.591	0.628
capillary height *h* (µm, assumed)	50	50

**Figure 4.**
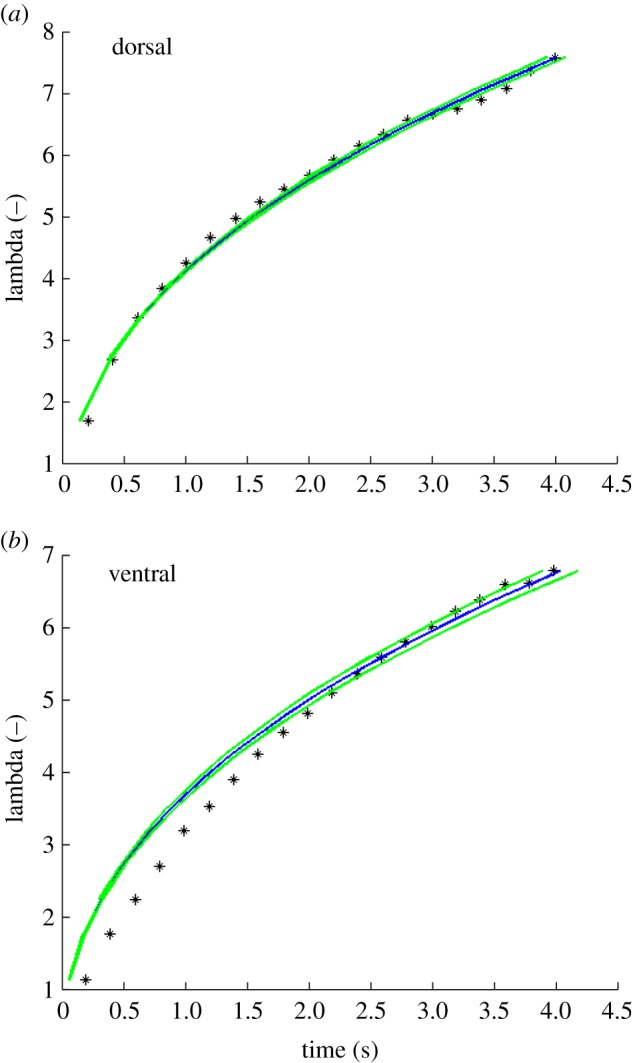
Water transport by the skin of *Moloch horridus* modelled with the dynamics function after Chandra and Yang [37], i.e. equation (3.2). Lambda is the dimensionless transport distance, with measured data (black), corresponding fit (blue) and 95% confidence interval (green). (*a*) Dorsal surface, (*b*) ventral surface. The fit parameters were determined as: *ϕ* = 0.599, *θ* = 44.38° (*a*) and *ϕ* = 0.630, *θ* = 45.92° (*b*).

Modelling the data reflected the measured values (figure 4). Most input parameters were measured and the modelled contact angles were in the typical range of skin contact angles for both body sides, i.e. *θ* = 44.38° (dorsal) and *θ* = 45.92° (ventral). The obtained values of the projected scale area fraction *ϕ*, i.e. *ϕ* = 0.599 (dorsal) and *ϕ* = 0.630 (ventral), were close to those calculated from light microscopic measurements of scale size (dorsal: 0.591, ventral: 0.628; table 3) (figure 4).

## 4. Discussion

We found for the thorny devil, as has been previously reported [19,21,23,25], that water is transported in channels between the scales rather than simply spread over the skin surface, as most of the scale surfaces are not covered by water during transport (figure 1). The transport parameters of single water droplets, i.e. velocity, transport duration and covered area, do not differ between dorsal and ventral body surfaces. Water transport capacity could be divided into two phases, which correlates with morphological structures; phase 1 is from application of a water droplet to it becoming incorporated (soaked) into the skin (i.e. from *t*_0_ to *t*_1_), and phase 2 until when the soaked droplet stops spreading, (i.e. from *t*_1_ to *t*_2_). No directional transport of water droplets was observed for thorny devil skin, unlike for the Texas horned lizard *Phrynosoma cornutum* and the Horvath's toad headed agama *Phrynocephalus horvathi* [19,30,38].

### 4.1. Capillary filling during water transport

When water droplets are completely adsorbed (soaked) into the capillary channels at *t*_1_, i.e. after about 2 s, the channels can be considered to be entirely filled. As transportation continues significantly thereafter, resulting in greater area covered and thus more capillaries included, it seems logical that the channels within the spreading area can now only be filled partially. Hence, we calculated the proportion of applied water volume to the theoretical channel volume at the time that the droplet is adsorbed (*t*_1_) and the time when transportation stops at *t*_2_.

Based on gross observations and SEM analysis of the skin and shed skin layers, the skin channel system can be generalized as a hexagonal capillary network (figures 1 and 2). For an ideal hexagon pattern (depicted in figure 2*d*), each edge (i.e. capillary) is neighbour to 2/6 of the hexagonal circumference, hence to 1/3 of the corresponding area. Then, the mean length of channels can be calculated geometrically from measured number of channels per area as 461.0 ± 38.5 µm (dorsal) and 474.3 ± 30.7 µm (ventral). This is more than the length of longitudinal channels measured from SEM analysis of shed skin layers for dorsal side (366.9 µm, *t*-test: *p*_(dorsal)_ = 0.044) but statistically the same as for ventral side (485.8 µm, *t*-test: *p*_(ventral)_ = 0.745). The precise cross-sectional shape of the channels can be neglected in calculating the volume of the channels. Hence, we determined the average cross-sectional area by the total length of channels involved at the time the applied droplet of 7 µl is soaked into the channels (i.e. at *t*_1_) as 22 477 ± 1408 µm^2^ (dorsal) and 21 834 ± 2749 µm^2^ (ventral). Compared to simple geometric shapes, this would correspond to particular radii or edge lengths (table 4). The calculated proportion of filled channel volume at the time the droplet is adsorbed into the skin at *t*_1_ is assumed to be 1.00, and when transportation stops (i.e. *t*_2_) it is 0.46 ± 0.02 for dorsal and 0.55 ± 0.04 for ventral body side (no significant difference; *t*-test: *p* = 0.06). Such half-filling of the capillary channel system seems to correspond with the morphology of skin channel structures.

**Table 4.** Calculated capillary cross section from water droplet volume and capillary length at time *t*_1_, and corresponding radius or edge length for round or square capillaries, respectively. Calculation based on capillary length of 461 µm (dorsal) and 474 µm (ventral), number of capillaries (see text) and 7 µl droplet volume. Data are mean ± s.e.m.

body side	cross section (µm^2^)	radius (µm)	edge length (µm)
dorsal (*n* = 22)	22 477 ± 1408	83.8 ± 2.5	148.6 ± 4.5
ventral (*n* = 19)	21 834 ± 2749	80.8 ± 4.9	143.3 ± 8.7

### 4.2. Potential influence of channel morphology on water transport

The 50% filling of the channel system at the time that transportation ceases is potentially related to particular morphological structures. Numerous protrusions into the channel cavities have been described as surface enlargements in a previous study [23]. We found that such protrusions form a sub-channel structure, i.e. smaller capillaries within the main channels (figure 3), and estimated the volume of these sub-channels to be about 50% of total channel volume. The width of sub-channels was estimated as about the same as the distances between two inversions from the inside view of shed skin layers, i.e. 19.1 µm (dorsal) and 26.4 µm (ventral). This is smaller than the up to 50 µm reported for *Moloch* [35] and *Phrynosoma* [23].

As capillary forces are inversely proportional to the capillary diameter, smaller channels can transport water over a greater distance (or height). Hence, we suggest that there is an important functional role of the sub-channel space for water transport, and the hierarchical capillary systems appear to be an adaptation for moisture-harvesting lizards such as *M. horridus* and *P. cornutum*. We suggest that the large capillary spaces between the scales quickly adsorb water into this dendritic hexagonal capillary system, and the sub-channel spaces transport water over greater distances. We calculated this extension in transport distance to be about 39%, making the sub-channel structure a significant distance extender for transport of water over the skin.

We calculated the channel volume as 5.76 µl cm^−2^ (dorsal) and 4.45 µl cm^−2^ (ventral), which reflects the significant differences in channel width (dorsal: 186 µm, ventral: 158 µm). However, the difference in width of sub-channels was reversed (dorsal: 19.1 µm, ventral: 26.4 µm). Considering that sand shovelling behaviour has been observed in a previous study [21], it seems likely that these morphological differences are minor adaptations to water uptake and transport. A ventral contact to moist sand could require smaller main capillary channels to overcome the sand water potential, whereas gravity can be used for sand water uptake on dorsal body surface which may only require larger main capillaries for transportation, but smaller sub-channels for distance extension.

### 4.3. Differences between live and preserved thorny devils

Water transportation by skin of live *Moloch horridus* was characterized by durations between 8.6 ± 0.8 s (ventral) and 9.8 ± 1.0 s (dorsal) and no delay, i.e. all water droplets were transported immediately after application. Compared to live specimens, durations for the skin of preserved specimens were significantly longer for dorsal side (29.5 ± 15.3 s, Mann–Whitney *U* test: *p* = 0.038), whereas no difference was found for ventral skin (11.2 ± 5.4 s, Mann–Whitney *U* test: *p* = 0.363). However, other than these quantitative differences, water transport for live and preserved specimens was in principle similar. The quantitative differences can possibly result from the alcohol used for preservation. Although specimens were dried for about one hour, an alcohol residue might remain in the channels, and as any residual alcohol can be neither quantified nor controlled, its influence can only be speculated upon. We would expect residual alcohol to facilitate capillary uptake and distribution of water, because of the lower surface tension of alcohol than water. Furthermore, the alcohol storage could remove cutaneous lipids and other molecules [39], and this might alter the wettability of the skin keratin layer and hence water transport. However, such removal/altering of molecules appeared very slow as no effects of preservation of shed skin layers could be observed (unpublished observation). Thus, although precise measurement of the transport properties of the skin requires live specimens, data from preserved lizards can still be useful as an indicator of a possible, but non-quantitative, role of the skin for moisture-harvesting.

### 4.4. Modelling of skin water transport

Transportation of droplets applied to the lizard's skin continued significantly after complete adsorption of the droplet. This continued transportation could result from the sub-channel structure, which is considered to enhance the transport distance based on greater capillary forces. The abstracted skin morphology of thorny devils describes hexagonal pillars (i.e. scales) surrounded by a network of capillary channels. Similar systems have been modelled with a Washburn dynamics function (transport distance ∼ time^0.5^ [40,41]). However, the Washburn model had been found to overestimate the transport velocities for surface channel structures formed by pillars. The dynamics function of Chandra and Yang [37] contributes stronger to geometrical surface parameters and hence friction forces (equation (3.2)) to overcome the limitation of the Washburn model [37].

Taking such potential major influence of sub-channels into account, a system with principally constant water source could be a reasonable contribution to the obtained transportation properties [37]. Applying this dynamic yielded reasonable fit parameters, i.e. contact angle *θ* and fraction of projected scale basis area *ϕ*; measured and fitted parameters were very similar and contact angles of about 45° are likely to reflect the wettability of thorny devil skin as described previously [19]. Most input parameters were measured and those modelled were in the typical range of thorny devil skin. Therefore, we suggest that water transport by the skin of thorny devils can be modelled by the dynamics function of Chandra and Yang [37], showing that wetting effects have a dominant influence on skin water transport.

## 5. Conclusion

The ability to transport water using the integument appears to be a major adaptation of thorny devils to their habitat. The complex nature of skin channels itself suggests an important adaptational role of integumental water transport, along with the remarkable convergence of skin structure and function in this regard for *Moloch horridus* and *Phrynosoma cornutum*. Skin water transport of thorny devils was symmetric (unlike for *P. cornutum*), i.e. the applied 7 µl water droplets were transported radially over more than 9.2 mm within about 10 s. This transport takes place within about 1600 channels of the hexagonal capillary network, each with an average length between 284 µm and 486 µm.

In addition to the two basic biophysical/morphological requirements of moisture harvesting, i.e. hexagonal channel network between the scales and their micro-ornamentation, we describe how these channels are hierarchically structured. The division of the large channels into smaller sub-channels is proposed as a further structural adaptation. Large channels between the scales can quickly adsorb water into their high-volume channel system. The sub-channel structures, formed by protrusions within the larger cavities, appear to act as distance extenders for capillary water transport by about 39%. Thorny devils would further benefit from water transport in hierarchically structured channels as this potentially reduces the volume of water required for drinking. Hence, we propose that the sub-channel structure is an important morphological adaptation.

The complex skin morphology of thorny devils was modelled as a surface channel structure formed by pillars. A system with a principally constant water source was found to be a reasonable contribution to the obtained transportation properties. We showed that the water transport by the skin can be modelled using a dynamics function by Chandra & Yang [37], for which most morphological input parameters could be measured.

Unfortunately, we have shown that the quantitative measurement of skin water transport and the ability to harvest moisture with the integument requires live specimens, precluding the study of museum-preserved specimens for detailed and quantitative aspects of cutaneous water transport, although the basic morphological requirements for skin water transport are still apparent.

## Supplementary Material

Thorny devil water transport figure S3

## Data accessibility

Data are available in the electronic supplementary material.
