# Supplementary material for: Adsorption and movement of water by skin of the Australian thorny devil (Agamidae: Moloch horridus)
Source: R Soc Open Sci. 2017 Sep 13;4(9):170591. doi: 10.1098/rsos.170591 (PMC5627102; doi:10.1098/rsos.170591)
Supplement: Thorny devil water transport figure S3 [file rsos170591supp1.pdf]

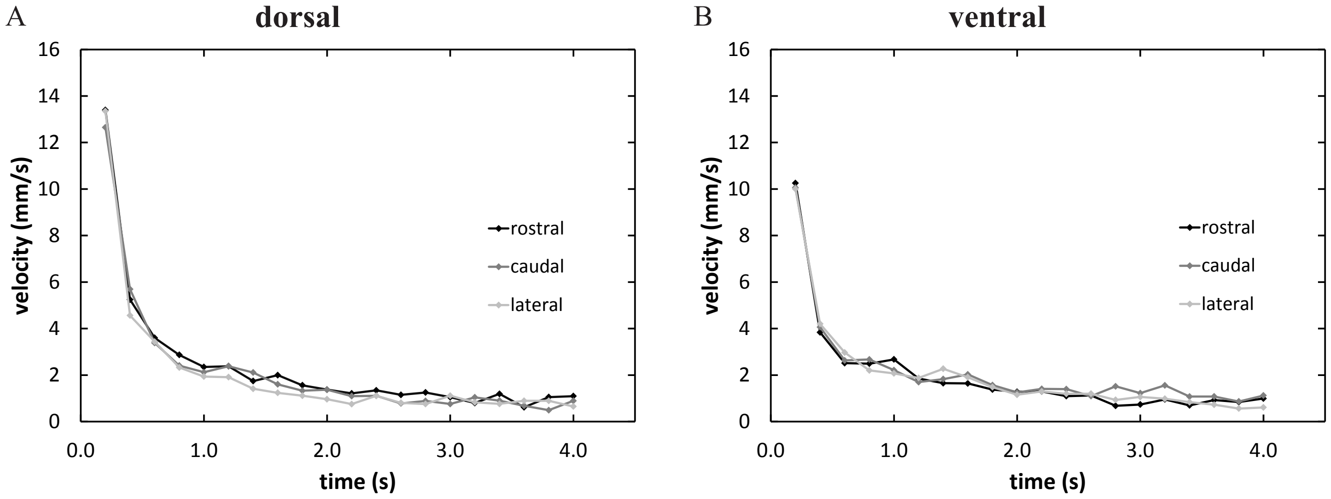

Figure S3: Transport velocities of single droplets applied onto the skin of *Moloch horridus*. Transport was characterised by direction: rostral (black), caudal (grey) and the average of lateral (light grey). A) Dorsal. B) Ventral.
